# The Common Task

**Published:** 1907-05-18

**Authors:** 


### THE COMMON TASK.

### The Care of the Milk in Institutions.

The measures recommended by the Special Committee of the Royal Institute of Public Health for ensuring a clean milk supply, would, if carried into effect, probably abolish altogether the necessity for sterilisation. They relate to the maintenance of a healthy stock of cows, hygienic premises, cleanliness on the part of the milker, prompt refrigeration, and careful transport. It is disheartening to know that legal control over the milk trade is already in the hands of the local authorities, by means of the Order of 1885, but that this Order is in most districts a dead letter owing to the supineness of the local sanitary authority. What is needed, undoubtedly, is that the Order, or better still the Order amended as recommended by this Committee, should be rendered compulsory, and not permissive. It is astonishing that while public attention is repeatedly called to the filthy conditions under which the average dairy farmer carries on his business, so little effort should be made by those on whom the duty of regulating the trade rests to cope with the notorious abuses to which it is exposed. The proposed registration of dairymen is essential to any real control over their premises. It will take a vast deal of rigid inspection to educate the ordinary farm-hand into notions of cleanliness in the milking process, and we believe that the provision of proper lavatory accommodation in every milking shed for the ablutions of the milker, who, it must be remembered, is commonly the person responsible for cleaning out the stables, is an important point. This is a detail which has escaped the attention of the Committee, but unless the labourer has the washing made very easy for him, no amount of regulations will induce him to wash. With regard to means of transport, can no substitute be found for the ordinary pewter churn? The battered and dirty appearance of these vessels, both outside and inside, whether dirty or clean, seems to make the rinsing process a veritable work of supererogation. Even in hospitals where all else is shining with elbow grease, the milk cans, unless newly bought, are conspicuous for their dinginess. The practice of bottling the milk and despatching it packed in cases, which has been found practicable on the Continent and in America would be an ideal system for the institution, and would obviate all possibility of contamination.

### Nursing Without a Nurse.

There are still many people who for want of means, or through poverty, are compelled to nurse their sick relatives by the light of their own intelligence and the doctor's directions. For such, the little book on "Sick Nursing," by H. Drinkwater, M.D. (Dent and Co., 1s. net) will prove a great source of enlightenment and cheer. Intended primarily for the use of students in connection with St. John's Ambulance classes, it is well fitted to be a guide to the public in such matters of health and sickness as concern the laity. We think it might be useful for the relatives in many cases of illness, even when the help of a trained nurse can be obtained, since the reason for measures they see the doctor and nurse carrying out are concisely stated, without the smallest digression into the sphere of matters purely medical. In this way nurses will find it a useful auxiliary with "the family."

### The Hospital Balcony.

The balcony as an adjunct to hospital treatment has hardly yet met with sufficient recognition at the hands of the architects. It is seen to full advantage at Addenbrooke's Hospital, where the noble proportions of the wards are enhanced by the colonnades extending along the façade, and affording the best possible opportunity for open-air treatment. Here, summer and winter alike, looking over the lawns of the hospital to great elms in the background, where rooks are perpetually busy, the patients lie accommodating themselves easily to changes of temperature and imbibing such a taste for air as shall revolutionise their homes from that time on. It is not only phthisical patients who respond to this treatment. The outdoor section of the surgical ward is a strong feature of the hospital; there is space for twelve beds, six male and six female, under the broad colonnade which lines these wards; the effect upon obstinate wounds and ulcers is eminently satisfactory, and obviates the difficulty of finding convalescent places for such patients. An outdoor annexe to a ward undoubtedly adds much to the responsibilities of the nurse. She cannot keep the whole ward under supervision at the same moment, and must be perpetually on guard lest any of her outdoor patients, especially the new ones before they are acclimatised, are exposed to serious discomfort. Besides, the weather requires a certain amount of watching, for scuds of rain on that side must be guarded against with screens or waterproof blinds. It may be assumed, however, that whatever tends to widen the duties of the hospital nurse, adds to the value of the training received. And experience in the management of open-air patients is a very valuable asset for the private nurse. The continual development of open-air treatment, both in surgical and medical cases, renders it imperative that the nurse should understand its peculiarities, and be trained in the exercise of tact in carrying it out. And this may be more suitably done in a hospital where it forms part of the regular work than in sanatoriums where, as a rule, only mild cases are received. If the town and county of Cambridge could only awake to a full perception of the work which is being carried out under their eyes as they pass to and fro, the pressure of financial care would speedily be lifted from the shoulders of the hospital authorities.

### Premiums for Probationers.

We note that the entrance fee for probationers at the Royal Portsmouth Hospital is £5, and not £1 1s. as stated.

